# An Exploratory Study on Vectorcardiographic Identification of the Site of Origin of Focally Induced Premature Depolarizations in Horses, Part I: The Atria

**DOI:** 10.3390/ani12050549

**Published:** 2022-02-23

**Authors:** Glenn Van Steenkiste, Tammo Delhaas, Ben Hermans, Lisse Vera, Annelies Decloedt, Gunther van Loon

**Affiliations:** 1 Equine Cardioteam, Department of Internal Medicine, Reproduction and Population Medicine, Faculty of Veterinary Medicine, Ghent University, 9820 Merelbeke, Belgium; lissevera@hotmail.be (L.V.); annelies.decloedt@ugent.be (A.D.); gunther.vanloon@ugent.be (G.v.L.); 2 Department of Biomedical Engineering, CARIM School for Cardiovascular Diseases, Maastricht University, 6200 MD Maastricht, The Netherlands; tammo.delhaas@maastrichtuniversity.nl (T.D.); ben.hermans@maastrichtuniversity.nl (B.H.)

**Keywords:** focal, atrial tachycardia, P wave, electrophysiology, electrocardiography

## Abstract

**Simple Summary:**

Arrhythmias are common in horses, but in order to offer adequate treatment, the anatomical location from where the arrhythmia originates must first be known. The objective of this study was to differentiate between the various anatomical origins of atrial premature depolarizations and to further differentiate these from normal sinus rhythm based upon the vectorcardiography characteristics of seven horses without cardiovascular disease. With vectorcardiography, the magnitude and direction of the cardiac electrical forces were derived from an electrocardiographic recording and were plotted in three dimensions. The three directions were represented by the right–left axis, head–tail axis, and dorsoventral axis. Under general anaesthesia, atrial premature depolarizations were induced by pacing inside the heart, guided by a 3D mapping system that allowed us to see the 3D position of the pacing catheter inside the heart in real-time. An adapted statistical test optimized for spherical data was used to demonstrate that the maximal directions of the first and second half of the P wave showed significant differences between the paced locations and between the paced complexes and sinus rhythm. The current study provides a practical approach to identifying the approximate site of origin of an atrial arrhythmia using a custom 12-lead ECG.

**Abstract:**

In human cardiology, the anatomical origin of atrial premature depolarizations (APDs) is derived from P wave characteristics on a 12-lead electrocardiogram (ECG) and from vectorcardiography (VCG). The objective of this study is to differentiate between anatomical locations of APDs and to differentiate APDs from sinus rhythm (SR) based upon VCG characteristics in seven horses without cardiovascular disease. A 12-lead ECG was recorded under general anaesthesia while endomyocardial atrial pacing was performed (800–1000 ms cycle length) at the left atrial free wall and septum, right atrial free wall, intervenous tubercle, as well as at the junction with the cranial and caudal vena cava. Catheter positioning was guided by 3D electro-anatomical mapping and transthoracic ultrasound. The VCG was calculated from the 12-lead ECG using custom-made algorithms and was used to determine the mean electrical axis of the first and second half of the P wave. An ANOVA for spherical data was used to test if the maximal directions between each paced location and the maximal directions between every paced location and SR were significantly (*p* < 0.05) different. Atrial pacing data were not available from the LA septum in three horses, the intervenous tubercle in two horses, and from the LA free wall in one horse. The directions of the maximal electrical axes showed significant differences between all paced locations and between the paced locations and SR. The current results suggest that VCG is useful for identifying the anatomical origin of an atrial ectopy.

## 1. Introduction

Atrial tachycardia (AT) in horses can be treated by a recently developed novel technique, i.e., ablation in which the focus of the atrial tachycardia is cauterized [1,2]. To shorten anaesthesia time and simplify the procedure of this advanced technique, accurate pre-procedural prediction of the anatomical origin of the atrial tachycardia by analysing the surface electrocardiogram (ECG) is advisable. In human medicine, specific 12-lead ECG characteristics have been described to differentiate between sinus rhythm (SR) and AT, as well as to identify the origin of atrial premature depolarizations (APDs) and AT [3,4]. Since previous studies in horses revealed contradictory results concerning the diagnostic value of the 12-lead ECG for the localization of premature depolarizations [5,6,7,8], the 12-lead ECG has been considered unreliable in horses. However, this unreliability might be related to the shortcomings of the applied lead systems. Indeed, guidelines for the electrode placement of a 12-lead ECG in horses were based on human electrode placement [9]. By placing the limb electrodes on the extremities of the horse, not only is Wilson’s central terminal (WCT) placed outside the heart, but Einthoven’s triangle is also almost perpendicular to the electrical axis of the equine heart, as well as ventral to the heart. This gave rise to unreliable results because of the wide individual variations of the recorded ECG trace between horses [10,11]. Moreover, the ECG was extremely sensitive to forelimb movement. Different orthogonal lead systems were proposed to overcome this issue, and although these were more reliable, they were never widely used except in experimental setups, as they required purpose-built ECG recording devices [8,11,12,13]. Recently, an adapted 12-lead ECG configuration was proposed [14], which can be applied using human ECG recording devices. This new electrode configuration addresses some of the original shortcomings by placing Einthoven’s triangle in a plane close to the mean electrical axis of the heart and the precordial electrodes in a plane perpendicular to the electrical axis of the heart [14]. Moreover, the configuration also allows the construction of vectorcardiograms (VCGs), in which cardiac electrical forces at a given point in time are summed into a single vector which has a direction and a magnitude. The past decades have shown a revival of vectorcardiography in human medicine owing to the possibility of mathematically synthesizing the three orthogonal leads of the VCG from the 12-lead ECG, avoiding the need for specialized recording equipment [15]. Whereas a 12-lead ECG is usually interpreted by a human physician from their knowledge of the different ECG complexes, a VCG can give a visual 3D representation of the electrical activity in the heart without the need for memorizing all the different ECG configurations. Since a VCG is able to be calculated from a 12-lead ECG, but the inverse transformation does not result in a perfect 12-lead ECG [16], the VCG contains slightly less information compared to the 12-lead ECG from a purely theoretical point of view. Nevertheless, the VCG also contains additional kinds of information that remain unexplored in the 12-lead ECG. Examples of these are the true P wave axes in three dimensions and the vector loop inscription direction [15,17,18].

The objective of this study was to determine if VCG-derived P wave characteristics could be used: 1. to differentiate between SR and pacing-induced APDs; 2. to identify the anatomical origin of the pacing-induced APD.

## 2. Materials and Methods

### 2.1. Study Set-Up

A cross-sectional analytic study design was used. This research was approved by the ethical committee of the Faculty of Veterinary Medicine, Ghent University (EC2016/35), and animal care was provided according to their guidelines. Seven warmbloods aged approximately 12.5 years (5–20 years, median range), 163 cm (158–179 cm) at the withers, and 548 kg (420–706 kg) were used. Four of the horses were owned by the Faculty of Veterinary Medicine, whereas three horses were donated by their owners for the purpose of scientific research followed by euthanasia because of pre-existing orthopaedic problems. Horses were included in the study if their auscultation, biochemistry (electrolytes and cardiac troponin I), echocardiography, and ECG results were normal. Data from the currently described study were obtained at the same time as those from a previously described study [19].

### 2.2. Electrophysiological Study

**Figure 1:**
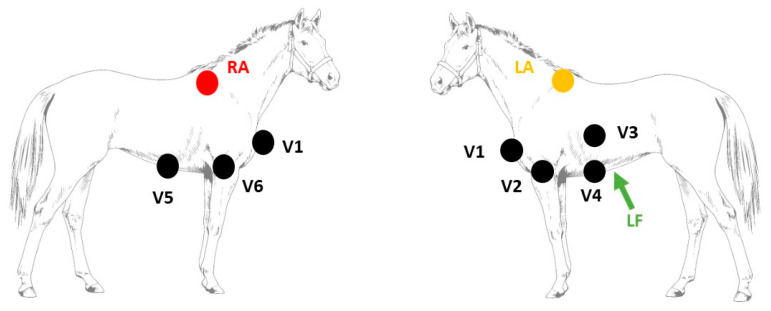
Electrode placement for the 12-lead electrocardiogram recording used in the current study [14]. Abbreviations: LA, left arm electrode; LF, left foot electrode; RA, right arm electrode; V, precordial electrode.

The study was performed under general anaesthesia in a position of dorsal recumbency. Prior to pacing, a complete endomyocardial 3D electro-anatomical map (Rhythmia v1.4, Boston Scientific, Diegem, Belgium) was created of the atria, as described elsewhere [20]. The 3D electro-anatomical map was used as a reference in order to navigate the mapping/pacing catheter (IntellaMap Orion, Boston Scientific, Diegem, Belgium) inside the atria to specific anatomical locations. Simultaneously performed echocardiography (Vivid 7, GE Healthcare, Diegem, Belgium) served as an additional confirmation of the pacing location. Supra-threshold pacing was performed at 800–1000 ms cycle length (EPS 320, Micropace EP Inc., Santa Ana, CA, USA) at the following 6 locations: the LA caudal free wall, septum, RA free wall, intervenous tubercle, the junction with the cranial (CrVC) vena cava, and the junction with the caudal (CaVC) vena cava. Sinus rhythm was included in the dataset as a 7th location of atrial activation origin. Twenty pacing-induced APDs for each location were recorded with a 12-lead ECG (Labsystem Pro v2.6, Boston Scientific, Diegem, Belgium) with the electrode configuration as shown in Hesselkilde et al. ([14]; see Figure 1): the left and right arm electrodes were placed on the left and right dorsal spina scapula, respectively; the left foot electrode was placed at the abdominal midline caudal to the xiphoid process. The precordial leads were placed on the manubrium sterni (V1), on the ventral part of the left (V2) and right (V6) triceps muscle, in the left 6th intercostal space at the level of the shoulder joint (V3) and the elbow joint (V4), and in the right 6th intercostal space at the level of the elbow joint (V5). With X as the right–left, Y as the cranial-caudal-cranial, and Z as the ventral-dorsal-ventral axes [12], the VCG leads X, Y, and Z were calculated from the 12-lead ECG using the following equations:

(1) $$X=\frac{V2+\;V4}{2}-\frac{V5+\;V6}{2}$$

(2) $$Y=\frac{V4+\;V5}{2}-\frac{V2+\;V6\;}{2}$$

(3) Z=−aVF

### 2.3. Data Analysis

**Figure 2:**
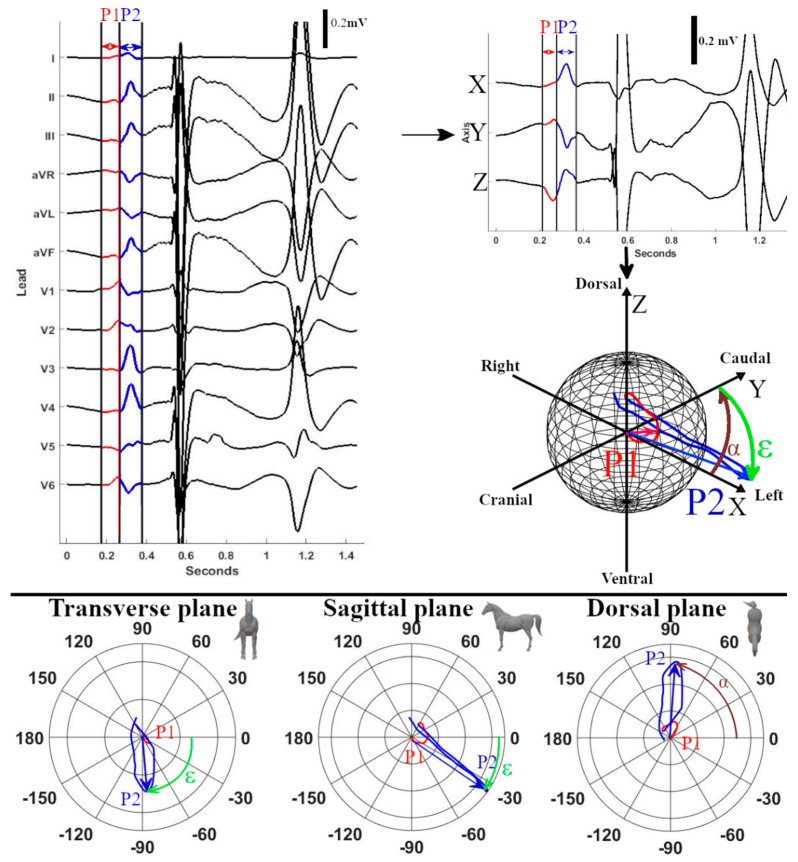
Example measurements of P wave characteristics of a median sinus rhythm or paced beat. The first (P1) and second (P2) part of the P (sinus rhythm) or P’ (paced complex) wave were selected on the 12-lead ECG. After selecting the timeframe, the VCG axes were calculated from the 12-lead ECG with X representing the right–left, Y the cranial–caudal, and Z the ventral–dorsal axes. After the calculation of the VCG, the spherical coordinates were calculated from the VCG; for P1 and P2, the coordinate with the largest radius is the mean electrical axis (MEA). The MEA is shown by the red (P1) or blue (P2) arrow on the sphere. The azimuth (α) and elevation (ε) of MEA_2_ are indicated on the sphere. Below the sphere, 3 polar plots are given with the transverse, sagittal, and dorsal projections of the VCG. The MEA of P2 is indicated on the polar plots by the blue arrow with the measurements for the azimuth and elevation. Note on the 2D polar plots that the elevation measured from the transverse plane has a different angle than the measurement from the sagittal plane. This is because the maximum radius of the VCG is view-dependent if projected into a 2D plane. It is also why the azimuth, elevation, and maximal radii for the statistics were measured in 3D space.

**Figure 3:**
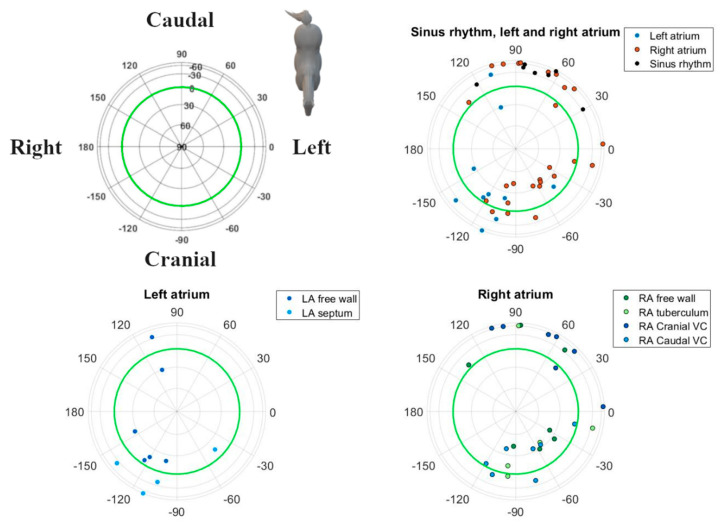
Spatial directions of the mean electrical axis (MEA) of the first half of the P wave for individual induced APDs visualized with a Lambert azimuthal equal-area plot. Dots inside the green circle represent a dorsal MEA, while dots outside represent a ventral MEA. Left is 0° and caudal is 90°. Different P wave origins are indicated by different colours. Abbreviations: LA, left atrium; RA, right atrium; VC, vena cava.

**Figure 4:**
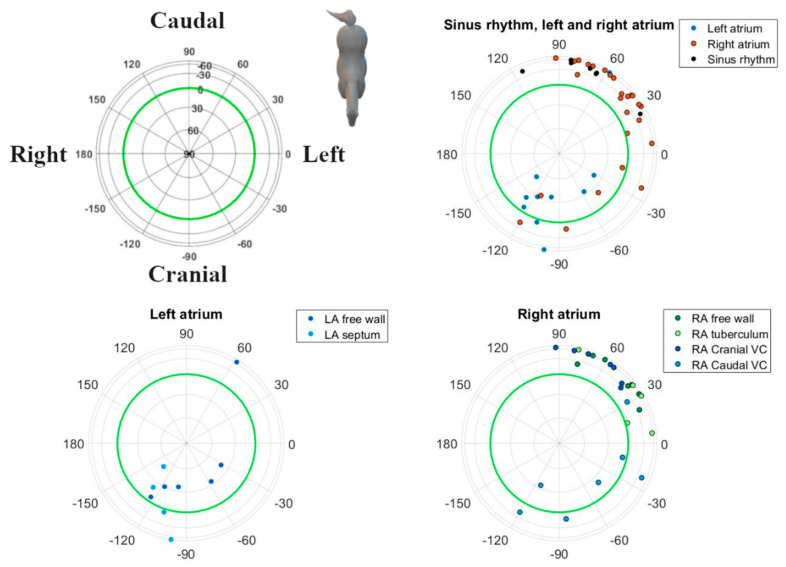
Spatial directions of the mean electrical axis (MEA) of the second half of the P wave for individual induced APDs visualized with a Lambert azimuthal equal-area plot. Dots inside the green circle represent a dorsal MEA while dots outside represent a ventral MEA. Left is 0° and caudal is 90°. Abbreviations: LA, left atrium; RA, right atrium; VC, vena cava.

Analyses were performed using custom-made programs (Matlab R2018b, MathWorks, Eindhoven, The Netherlands). Median complexes were calculated for each paced location and SR by taking the median value of each point in the ECG cycle over multiple consecutive complexes after alignment. Each complex was manually labelled by a single observer with thorough experience in equine electrocardiography. Unanalysable complexes due to noise and artifacts and complexes with a deviant morphology were excluded. Since in some cases, only 4 consecutive complexes were analysable, only 4 complexes were used for constructing the median complex. After alignment on the maximum absolute value of the P (sinus beat) wave and P’ wave (paced complex), the representative median complex was constructed for each lead using the median function of Matlab. For each horse, the onset, offset, and middle of the P’ wave and P wave for the median complex were manually annotated on the median vector amplitude by a single experienced observer. The P wave onset was defined as the initial deflection from the iso-electric baseline and the offset was defined as the junction between the end of the P wave and its return to the baseline prior to the QRS complex [21]. The middle was automatically defined as half of the total duration if the P wave was singular, or manually annotated as the notch if the P wave was bifid. The distance between the origin of the spherical coordinate system of the VCG, defined as the onset of the P wave, and a point on the VCG vector loop was taken as a measure of the amplitude. The vector amplitude was calculated using the Euclidean norm $\left|\left|V_{m}\right|\right|=\sqrt[{2}]{X^{2}+Y^{2}+Z^{2}}$. Peak amplitudes during the first and second half of the P wave (P1 and P2, respectively) were considered to be indicative of their spatial mean electrical axis (MEA_1_ and MEA_2_, respectively; Figure 2). The size and direction of both MEAs were automatically detected and expressed by the vector amplitude, azimuth (clockwise estimated angle between the projected vector in the X–Y plane and the positive X-axis), and elevation (angle between the vector and the X–Y plane with positive values denoting dorsally directed values). Lambert azimuthal equal-area plots were used to visualize the direction of the individual depolarizations (Figure 3 and Figure 4). The different steps of the analysis have been illustrated in Video S1.

### 2.4. Statistical Analysis

All statistics were done in R (R 3.6.1, R Foundation for Statistical Computing, Vienna, Austria). Azimuth, elevation, radius, and duration of MEA_1_ and MEA_2_ are described as mean ± standard deviation and with a 95% confidence interval. The Shapiro–Wilk normality test was used to test if the durations and radii were derived from a normal distribution. After normalization, the spherical coordinates were converted to a Cartesian coordinate system similar to that in Figure 2 in order to streamline the data analysis [22]. The function fishkent in the Directional package was used to test the null hypothesis that a von Mises–Fisher distribution, in which points are isotropically concentrated around a mean direction (rotational symmetry), would fit the data well. Rejection of the null hypothesis would imply a lack of rotational symmetry, in which case a Kent distribution would be more appropriate [23]. The mean direction and the concentration parameter κ of this direction were calculated for each paced location and SR [24]. An ANOVA for spherical data with post-hoc correction was used to test if the mean directions of each paced location and SR were significantly (*p* < 0.05) different from each other [25].

## 3. Results

**Figure 5:**
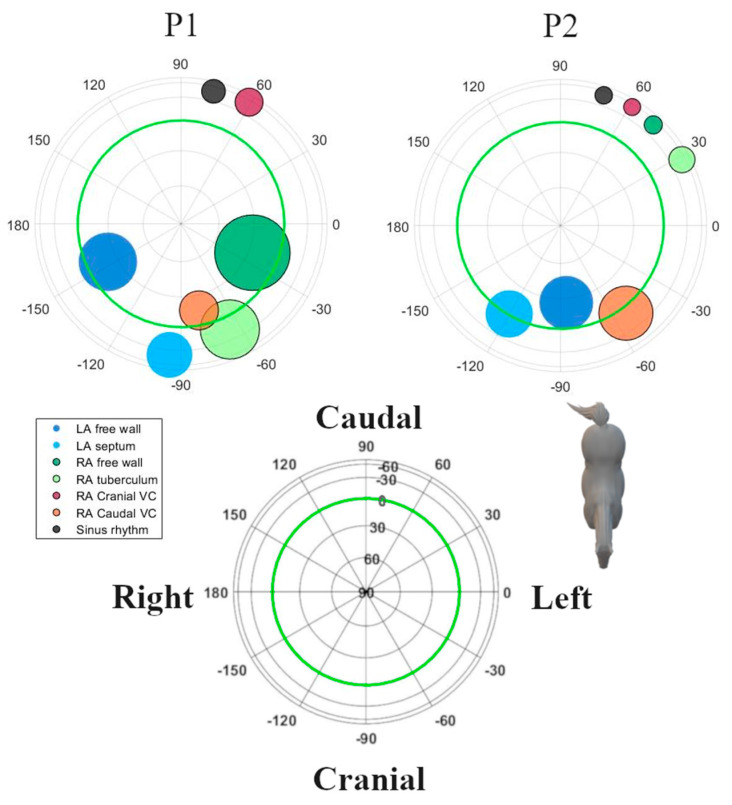
The mean spatial directions of the mean electrical axis (MEA) of the P’ wave for each location of induced APDs and for the SR visualized with a Lambert azimuthal equal-area plot. The radius of each circle is inversely related to the concentration parameter κ of each location with a smaller radius indicating less spreading between the horses of the individual MEAs originating from this location. Circles inside the green circle represent a dorsal MEA while dots outside represent a ventral MEA. Left is 0° and caudal is 90°. Abbreviations: LA, left atrium; P1, the first half of the P wave, P2, the second half of the P wave; RA, right atrium; VC, vena cava.

The ECG data quality was insufficient for analysis while pacing from the LA septum in three horses, the RA intervenous tubercle in two horses, and from the LA free wall in one horse. In these cases, due to the adjacent T wave or pacing spike, the P wave was partially hidden. In all other locations and during sinus rhythm, good quality ECGs could be recorded. One horse had two rate-dependent sinus node exit areas, one at the medial crista terminalis and another one at the right atrial free wall [19]. In this horse, the dominant P wave morphology correlating to the medial crista terminalis was selected for further analysis. Descriptive statistics for the P waves of the various atrial pace locations and the SR are given in Table 1. The durations and radii were all derived from a normal distribution. Distributions of the directions of MEA_1_ and MEA_2_ for the SR and each pace location showed rotational symmetry (*p* ≥ 0.05). Hence, a von Mises–Fisher distribution was assumed. The ANOVA showed that the directions of MEA_1_ (*p* < 0.001) and MEA_2_ (*p* < 0.001) were significantly different between all the paced locations as well as between the paced locations and the SR. Post-hoc results from the ANOVA for individual combinations are shown in Table 2. The mean direction and the concentration parameter κ of MEA_1_ and MEA_2_ for each location are shown in Figure 5. Only these mean directions are described below. For the individual variations of MEA_1_ and MEA_2_, the reader is referred to Figure 3 and Figure 4, in which the individual MEA_1_ and MEA_2_ are shown in the Lambert azimuthal equal-area plot.

In horses in SR, the average directions of both MEA_1_ and MEA_2_ were directed left caudoventrally. Pacing at the LA free wall induced a right MEA_1_ and a left cranial MEA_2_. Induced APDs from the LA septum had a right cranial MEA_1_ and MEA_2_.

For APDs originating from the RA to the CrVC junction, both MEA_1_ and MEA_2_ were directed left caudoventrally, whereas pacing from the RA to CaVC junction induced a left cranially directed MEA_1_ and MEA_2_. Four induced APDs from the RA free wall had a left cranial MEA_1,_ and three had a caudal MEA_1_. All APDs from the RA free wall had a left caudoventral MEA_2_. Pacing at the intervenous tubercle showed a left cranial MEA_1_ and right caudoventral MEA_2_.

## 4. Discussion

The current study describes the P wave characteristics during SR and pacing-induced APDs at different anatomical locations in an experimental study set-up. Based upon VCG characteristics, significant differentiation between sinus rhythm and APDs in general as well as between APDs from different anatomical locations was found.

Since the applied, recently described electrode placement [14] has the limb leads embracing the thorax, the Einthoven triangle is almost in line with the mean electrical axis of the equine heart, and the Wilson central terminal (WCT) is close to the heart. In combination with the unipolar precordial leads placed in a plane perpendicular to the mean electrical axis, more reliable information about atrial and ventricular electrophysiology could be obtained, as compared to older electrode placement configurations [9,14], which were based upon human electrode placement with limb leads on the limbs of the horse. These historical electrode placement configurations in horses with limb leads on the limbs recorded potentials in a plane perpendicular to the mean electrical axis, had the WCT far outside the heart and, hence, due to the wide individual variations in the recorded ECG traces between horses, gave rise to unreliable results. Moreover, the ECG was also extremely sensitive to forelimb position or movement [10,11].

Our electrode placement also allowed us to calculate electrical forces in three orthogonal, anatomically well-defined directions. These left–right, cranial–caudal, and ventral–dorsal leads were used to reconstruct the VCG. Based on this VCG, we were able to derive the following information. During SR at rest, P1 was generated by the depolarization of the RA in a caudoventral direction; P2 was produced by the depolarization of the LA, again in a caudoventral direction, as described previously [26]. The SR VCG is similar to the APDs originating from the junction between RA and CrVC, making it difficult to distinguish between both, which is also the case in humans [27]. One horse had a MEA_1_ in SR that was directed left craniodorsally. This particular horse had two sinus node exit areas, one at the medial crista terminalis and another at the right atrial free wall [19]. The P wave of the sinus rhythm originating from the RA free wall had a MEA_1_ which was close to a cluster of APDs originating from the RA free wall. Likewise, in humans and dogs, the exit area of the sinus node can vary, both within and between individuals [28,29,30]. In humans, the sinus node region has been described to be as large as 7.5 × 1.5 cm, and some preferential pathways of sinus node exits have been associated with atrial remodelling [31]. Table 2 and Figure 3 and Figure 5 show that the clusters of P1 represent the anatomical origin within the atria of the induced APD, which also allows for differentiation between the anatomical origins within the atria of the APD. Note that the cluster sizes of P1 and P2 for the LA free wall are very similar (Figure 5; Table 2) while there appears to be a greater difference between the P1 and P2 cluster sizes in Figure 3 and Figure 4. Despite offering accurate area representation in all regions of the sphere, the Lambert azimuthal equal-area projection does not accurately represent the angles. This leads to a distortion at the edges of the 2D representation of the sphere. The results from P2 show clustering representing the interatrial origin of the APD. This matches previous findings in horses [19,32]. Indeed, during P1, the depolarization wave spread within the atrium of origin, causing MEA_1_, while during P2, the depolarization spread to the adjacent atrium, causing MEA_2_. Since P1 resulted from the depolarization of the paced atrium, it is more sensitive to slight changes in catheter position during pacing than P2, which represents the depolarization from right-to-left or vice versa using well-defined pathways. This could explain the large variation in results for the LA free wall MEA_1_, as manoeuvring the catheter to a specific location was more difficult. Figure 3, Figure 4 and Figure 5 show that the current results allow us to differentiate between a left or right atrial ectopic rhythm, but the prediction of where the origin lies within an atrium might be limited to larger areas. The induced APDs of the RA free wall had a left craniodorsal MEA_1_ in four out of seven horses, whereas in the remaining three horses, these RA free wall-induced APDs had a caudoventral MEA_1_. This could be explained by slight differences in the free wall pacing site. The presence of the left cranial MEA_2_ of APDs originating from the junction with the CaVC is in contradiction with a previous study [32] in which APDs induced at the ostium of the coronary sinus also had a cranially directed MEA_1_ but were followed by a caudally directed MEA_2_. In our results, MEA_2_ for the RA to the CaVC junction was in a left cranial direction in most cases, which implies that the LA is also depolarized from caudal to cranial. This makes it difficult to differentiate between the RA to CaVC junction and the LA. Similar to in humans, in which an anterior breakthrough site from the right to the left atrium through the oval fossa or coronary sinus has been describedKlik of tik om tekst in te voeren., we found evidence for this same breakthrough site in horses [19,33,34]. Another possible explanation for the left cranial orientation of MEA_2_ while pacing near the CaVC might be that pacing at this site resulted in the transmural electrical stimulation of the adjacent left atrial myocardium. The unexpected caudally directed MEA_2_ in two horses while pacing at the LA could be caused by a left-to-right atrial breakthrough site at the Bachmann bundle, while the cranially directed MEA_2_s could have a caudal left-to-right atrial breakthrough site. In human medicine, left-to-right breakthroughs have been described at the level of the oval fossa, coronary sinus, and the intervenous tubercle at the insertion point of the Bachmann bundle [35]. Whatever the underlying mechanisms of the breakthrough from RA to LA or from LA to RA in horses, they can be elucidated by the electro-anatomical mapping of the atria while pacing at the RA or LA.

Human 12-lead ECG characteristics [36,37,38,39] cannot be applied in horses because of the different anatomical positioning and electrophysiology of the equine heart. No direct comparison of the current data with the previously described P wave characteristics for horses was able to be made because of a different electrode configuration and the different anatomical locations for the origins of the APDs [10,32].

### Limitations

Only a small number of animals was used in the current study, but the power of the test was sufficient to show that the VCG derived from a 12-lead ECG has an added value for equine electrocardiography and can be used to identify the anatomical origin of atrial ectopy. Though only four complexes could be used to construct the median complex, instead of the planned twenty complexes, we observed no visual differences between the median complexes constructed from either four or twenty complexes in the cases with twenty complexes available. The current study was done in healthy horses and in an experimental design with induced APDs. However, similar pacing studies in humans have been useful in defining the diagnostic criteria applicable to clinical patients [37]. Nevertheless, more studies should be done in a larger group of horses, preferably standing, in order to account for more possible anatomical variations between animals. Some structural abnormalities, e.g. small areas of fibrosis, could have been missed with the current inclusion criteria, with a possible impact on the resulting vectors. Some anatomical locations, such as the LA septum and the RA intervenous tubercle, were only available from a limited number of horses because the recordings taken of the other horses had poor ECG quality. This might have impacted the statistical power of our test for these locations.

Although improved, the current electrode configuration might still not be the most optimal, because the WCT is still dorsal to the heart instead of in the centre of the heart. However, because of the current method of calculation of the VCG from the 12-lead ECG, the effect of WCT is completely omitted from all axes, since X, Y, and Z are bipolar. Most of the precordial electrodes were placed in a plane near the apex of the ventricles, while in human and small animal medicine, the electrodes are placed as close as possible to the middle of the heart in order to obtain better sensitivity and specificity in detecting a change in the conduction pattern.

## 5. Conclusions

The current results suggest that the VCG derived from a 12-lead ECG is useful in identifying the anatomical origins of an atrial ectopy. This VCG has the potential to be useful in the construction of a clinically applicable algorithm for the purpose of differentiating between the varying anatomical origins of APDs. Further research should be done not only in a larger group of horses with experimentally induced arrhythmias, but also in clinical equine patients, in which the underlying causes and locations of APDs or AT are identified using endocardial mapping. Alternative electrode positions should also be investigated.

## Figures and Tables

**Table 1:** Descriptive statistics of the electrical axis distributions for the different induced atrial premature depolarizations. The mean electrical axis is measured as the maximum radius during the first (P1) or second (P2) half of the P wave.

Pacing Location	*n*	Mean Electrical Axis P1					Mean Electrical Axis P2				
		Azimuth (°)	Elevation (°)	Radius (mV)	Duration (ms)	κ	Azimuth (°)	Elevation (°)	Radius (mV)	Duration (ms)	κ
Sinus rhythm	7	69 ± 48	−44 ± 14	0.10 ± 0.05	70 ± 20	12	72 ± 25	−47 ± 6	0.18 ± 0.05	80 ± 20	21.2
**Left atrium**											
Free wall	6	−156 ± 66	11 ± 21	0.14 ± 0.07	100 ± 30	1.9	−77 ± 62	18 ± 36	0.18 ± 0.09	90 ± 20	2.3
Septum	4	−102 ± 34	−31 ± 37	0.11 ± 0.07	50 ± 10	3.1	−117 ± 14	1 ± 50	0.20 ± 0.08	80 ± 10	3.0
**Right atrium**											
Caudal vena cava junction	7	−78 ± 35	12 ± 21	0.13 ± 0.08	100 ± 30	4.3	−53 ± 54	−4 ± 24	0.13 ± 0.06	80 ± 10	2.3
Cranial vena cava junction	7	61 ± 32	−51 ± 23	0.09 ± 0.05	90 ± 30	8.4	63 ± 18	−51 ± 14	0.24 ± 0.09	90 ± 10	22.6
Free wall	7	−16 ± 78	11 ± 37	0.13 ± 0.08	90 ± 40	1.2	47 ± 18	−47 ± 13	0.21 ± 0.08	90 ± 20	20.1
Intervenous tubercle	5	−55 ± 69	−9 ± 35	0.15 ± 0.14	80 ± 40	1.9	72 ± 25	−50 ± 23	0.18 ± 0.09	80 ± 30	9.6

κ, concentration parameter; *n*, number of horses.

**Table 2:** Post-hoc results for the ANOVA for each combination of induced atrial premature depolarizations and for each combination with sinus rhythm. The ANOVA was done based upon the first and second maximum electrical axis of the P wave. A value of *p* < 0.05 is considered as significant and is shown in bold.

	Left Atrium		Right Atrium			
	Free Wall	Septum	Free Wall	Intervenous Tubercle	Caudal VC	Cranial VC
**MEA 1**						
LA septum	**0.010**					
RA free wall	**0.031**	**0.046**				
RA tuberculum	0.070	0.140	0.133			
RA caudal VC	0.078	0.098	0.133	0.141		
RA cranial VC	**0.004**	**0.023**	**0.049**	**0.035**	**<0.001**	
Sinus rhythm	**0.007**	**0.018**	0.057	**0.030**	**<0.001**	0.143
**MEA 2**						
LA septum	0.057					
RA free wall	**<0.001**	**<0.001**				
RA tuberculum	**0.001**	**0.001**	0.134			
RA caudal VC	0.137	0.114	**0.002**	**0.033**		
RA cranial VC	**<0.001**	**<0.001**	0.143	0.137	**0.005**	
Sinus rhythm	**0.001**	**0.001**	0.136	0.120	**0.006**	0.143

Abbreviations: LA, left atrium; MEA, maximum electrical axis; RA, right atrium; VC, vena cava.

## Data Availability

The data is available from the authors upon reasonable request.

## Supplementary Materials

The following are available online at https://www.mdpi.com/article/10.3390/ani12050549/s1, Video S1: Video illustrating the data analysis workflow. The video includes the annotation of the electrocardiogram (ECG), vectorcardiogram (VCG) calculation, unit sphere projection of the mean electrical axis, and transformation to a Lambert Azimuthal equal-area projection. Abbreviations: LA; left atrium; RA, right atrium; SR, sinus rhythm.
